# Exploring determinants of quality of life for virally suppressed people with HIV in Northern Taiwan

**DOI:** 10.1186/s12879-026-12591-5

**Published:** 2026-01-21

**Authors:** Hsin-Hao Lai, Yung-Feng Yen, Chien-Chun Wang, Tsen-Fang Yen, Po-Tsen Yeh, Su-Han Hsu

**Affiliations:** 1 Section of Infectious Disease, Taipei City Hospital Yangming Branch, Taipei, Taiwan; 2 School of Medicine, College of Medicine, National Yang Ming Chiao Tung University, Taipei, Taiwan; 3 Institute of Public Health, National Yang Ming Chiao Tung University, Taipei, Taiwan; 4 Section of Infectious Disease, Taipei City Hospital Heping Branch, Taipei, Taiwan; 5 Section of Infectious Diseases, Taipei City Hospital Linsen Chinese Medicine and Kunming Branch, Taipei, Taiwan; 6 Department of Nursing, Taipei City Hospital Linsen Chinese Medicine and Kunming Branch, Taipei, Taiwan; 7 Department of Pharmacy, Taipei City Hospital Yangming Branch, Taipei, Taiwan; 8 School of Pharmacy, College of Pharmacy, Taipei Medical University, Taipei, Taiwan; 9 Department of Exercise and Health Sciences, University of Taipei, Taipei, Taiwan; 10 1F., No. 105, Yusheng St., Shilin Dist, Taipei City, 111024 Taiwan (R.O.C.)

**Keywords:** Depression, HIV infection, Medication adherence, Public health, Quality of life, Taiwan

## Abstract

**Background:**

With the success of antiretroviral therapy (ART), quality of life (QoL) has become a central goal in HIV care under the “Fourth 90” framework. While Taiwan has made significant strides in achieving UNAIDS targets, QoL among people with HIV (PWH) remains an ongoing challenge. This study explored determinants of QoL among virally suppressed PWH in Taiwan.

**Methods:**

A cross-sectional study was conducted a major HIV clinic in Taipei, enrolling 767 virally suppressed PWH between December 2018 and June 2021. Viral suppression was defined as an HIV RNA level of < 40 copies/mL, quantified using a standardized RT-PCR assay, measured within 6 months prior to enrollment. QoL was assessed using the World Health Organization Quality of Life Scale – brief version (WHOQOL-BREF), depressive symptoms using the validated Center for Epidemiologic Studies Depression Scale (CES-D), and medication adherence using the Medication Adherence Report Scale (MARS-5). Sociodemographic and clinical characteristics were collected through structured interviews. Multivariable linear regression analyses were performed to identify factors associated with QoL domains.

**Results:**

Overall, 78.5% of participants reported good self-perceived QoL. Depressive symptoms were the strongest factor associated with lower QoL across all four domains (physical, psychological, social, and environmental), with reductions of approximately 2–3 points per domain. Higher income and educational attainment were associated with better QoL. Marriage and having a romantic partner were associated with higher psychological and social well-being.

**Conclusions:**

Among virally suppressed PWH in Taiwan, depressive symptoms, socioeconomic factors, and relationship status were strongly associated with QoL. These findings support the integration of routine depression screening, financial aid expansion, education-related support, and inclusive relationship policies into HIV care to advance the Fourth 90 goal of optimizing quality of life.

**Clinical trial number:**

Not applicable.

## Background

According to the Joint United Nations Programme on HIV/AIDS (UNAIDS) statistics [1], an estimated 39.9 million people were with HIV (PWH) globally in 2023, including approximately 6.7 million in the Asia-Pacific region. In Taiwan, reports from the Centers for Disease Control (CDC) indicated that, as of the end of March 2025, 36,188 PWH were still living [2]. To end this AIDS epidemic, the UNAIDS 90-90-90 targets, set for 2020, aimed to achieve: 90% of all PWH knowing their status, 90% of diagnosed individuals receiving antiretroviral therapy (ART), and 90% of those on ART achieving viral suppression. While this initiative led to substantial improvements in HIV care globally, it has since transitioned to the more ambitious 95-95-95 targets, aiming to accelerate efforts toward eliminating HIV/AIDS by 2030 [3]. With the development of effective ART and strategic interventions, HIV is increasingly viewed as a chronic, manageable condition rather than a terminal illness. PWH now have life expectancies approaching those of the general population [4]. However, despite Taiwan’s success in achieving high treatment coverage, aging PWH face additional health challenges. Prolonged ART use is associated with chronic conditions, such as coronary artery disease, osteoporosis, and cerebrovascular disease [5], which can significantly impact physical health and long-term well-being. While previous HIV management efforts primarily focused on viral control, the current paradigm must expand to address broader health and social determinants that influence quality of life (QoL) for PWH.

The “Fourth 90” framework, introduced by Lazarus et al. [6], expands the UNAIDS “90-90-90” targets by emphasizing the need to enhance QoL for PWH beyond achieving viral suppression. It recognizes that long-term survival alone does not guarantee well-being—ensuring physical, psychological, social, and environmental health is equally critical. Despite Taiwan’s progress in HIV control, stigma, discrimination, and social isolation remain major obstacles that significantly reduce QoL for PWH. These factors contribute to mental health issues, including depression, anxiety, and diminished self-esteem [7]. Additionally, HIV often disrupts employment stability, leading to financial difficulties, further exacerbating challenges in accessing healthcare and social support systems [8].

Since 2018, the number of newly reported HIV/AIDS cases in Taiwan has been on a decreasing trend, thanks to comprehensive strategies [9], which including the introduction of an HIV case management system in 2007, the adoption of single-tablet regimens as the preferred first-line therapy in 2016, the implementation of government-funded pre-exposure prophylaxis (PrEP) and the rapid initiation of ART within seven days of HIV diagnosis in 2018. Additionally, same-day ART after HIV diagnosis has been in place since 2021. These measures enabled Taiwan to achieve the Triple 90s in 2020 and make significant progress toward the Triple 95s in 2023, achieving 91-96-95.

While Taiwan has successfully reduced HIV transmission rates, QoL remains an ongoing challenge for PWH. Previous policies primarily focused on disease control rather than holistic patient well-being. To address these concerns, Taiwan introduced “The First Phase Plan to Eliminate HIV/AIDS by 2030”. These strategies include health education, universal testing, promoting updating evidence supporting the U = U (Undetectable equals Untransmittable) concept, and conducting training programs targeting various subgroups within multisectoral and community-based organizations (CBOs). Additionally, subsidies have been provided to HIV-designated hospitals and CBOs to develop group counseling or support group initiatives.

While these initiatives mark progress, gaps persist in mental health resources, social support networks, and long-term care services for aging PWH. Furthermore, Taiwan lacks sufficient research on QoL determinants specific to its sociocultural and healthcare context, reinforcing the need for localized studies.

Northern Taiwan, particularly Taipei, represents a unique sociocultural and healthcare context. The region hosts the country’s largest HIV treatment centers and concentrated case management systems, making it the primary hub for HIV care in Taiwan [9]. Epidemiological data from the Taiwan CDC indicate that approximately 40% of all PWH nationwide reside in northern Taiwan [2]. As the most urbanized area, Taipei reflects both advanced healthcare accessibility and persistent challenges such as stigma, discrimination, and limited mental health resources [10]. Moreover, while marriage equality has improved psychosocial well-being among sexual minority men [11], broader societal stigma toward PWH continues to shape quality of life outcomes across diverse populations. These dynamics underscore the need for localized investigation in northern Taiwan, where healthcare infrastructure and sociocultural factors intersect to influence QoL among PWH.

This study aims to explore the key determinants influencing QoL among virally suppressed PWH in northern Taiwan, focusing on physical, mental health, social and environmental dimensions. The findings will provide actionable recommendations for policymakers and healthcare providers to develop patient-centered strategies that align with the Fourth 90 framework, ensuring that PWH not only survive but also thrives with optimal health and well-being.

## Methods

### Study design

This cross-sectional study consecutively recruited virally suppressed individuals living with HIV from Taipei City Hospital’s largest HIV clinic, which serves as Taiwan’s primary HIV treatment center. Participants were enrolled between December 2018 and June 2021. Consecutive recruitment during routine clinic visits was applied to minimize selection bias.

The study was approved by the Institutional Review Board of Taipei City Hospital (approval number: TCHIRB-10612120). All participants provided written informed consent. This study was conducted in accordance with the principles of the Declaration of Helsinki.

### Participants

All individuals aged 18 years or older, living with HIV, virally suppressed and receiving regular follow-up care at the Taipei City Hospital (TCH) HIV clinic, were enrolled in this study after providing informed consent. Viral suppression was defined as an HIV RNA level of < 40 copies/mL measured within 6 months prior to enrollment. Written consent was obtained from each participant. A trained case manager conducted face-to-face interviews to administer the survey. Participants who completed the survey received a US$3 coupon as a token of appreciation for their time.

### Procedures

During the study’s enrollment period, participants who provided consent were interviewed face-to-face by a trained case manager. A standardized questionnaire was used to collect information on participants’ quality of life (assessed using the World Health Organization Quality of Life Scale – brief version [WHOQOL-BREF]), socio-demographic characteristics, recreational drug use, comorbid conditions, history of sexually transmitted infections (STIs), depressive symptoms (measured using the Center for Epidemiologic Studies Depression Scale [CES-D]), and adherence to antiretroviral therapy, evaluated through the Medication Adherence Report Scale (MARS-5).

The socio-demographic data included participants’ age, gender, sexual orientation, body weight, height, income, education level, living arrangements, and details of whom they shared their HIV status with. Sexual orientation was classified as homosexual or non-homosexual. The questionnaire also gathered details about recreational drug use, including methamphetamine, sildenafil, ecstasy, gamma-hydroxybutyrate (GHB), amyl nitrite, cannabis, heroin, cocaine, and alcohol. STI history included conditions such as syphilis, gonorrhea, and genital warts. Additional data were collected on participants’ use of single tablet regimens, and their HIV viral load and CD4 counts were measured within three months of enrollment.

### Measurement instruments

All instruments used in this study were previously published and validated questionnaires, including the WHOQOL-BREF [12], the CES-D [13], and the MARS-5 [14]. The WHOQOL-BREF questionnaire, a validated self-report tool, assesses physical health, psychological health, social relationships, and environmental well-being. The Taiwanese version, adapted for local populations, has demonstrated high reliability among PWH [12]. Each item is rated on a 5-point Likert scale, with scores summed to determine overall QoL. A cut-off score of ≥ 60 (based on self-perceived QoL and general health satisfaction) indicates good QoL, while scores < 60 signify poor QoL [15], we adopted a cut-off of ≥ 60 to represent “good” QoL. This cut-off was applied for descriptive interpretation of self-perceived quality of life rather than for diagnostic classification. Although this threshold was originally validated in a non-HIV population, it provides a pragmatic benchmark in the absence of established cut-offs for PWH. This definition was applied in Table 2 to facilitate interpretation of domain scores. In the present study, the internal consistency of the WHOQOL-BREF was excellent (Cronbach’s α = **0.920**).

**Table 1:** Characteristics of virally suppressed PWH and their quality of life

	Total participants, *n* = 767		WHOQOL-BREF domain, Mean ± SD							
			Physical		Psychological		Social		Environmental	
Socio-demographics	Mean	SD	Pearson correlation	*P*	Pearson correlation	*P*	Pearson correlation	*P*	Pearson correlation	*P*
** Age**, **yr**	37.9	8.89	0.017	0.632	0.031	0.393	-0.038	0.289	0.032	0.378
** Diagnosed**,** yr**	9.25	5.50	-0.039	0.279	-0.018	0.625	-0.025	0.497	-0.002	0.962
** Treatment duration**,** yr**	7.97	5.54	-0.012	0.747	0.013	0.713	-0.008	0.831	0.013	0.724
**Socio-demographics**	**n**	**%**	**Mean (SD)**	***P***	**Mean (SD)**	***P***	**Mean (SD)**	***P***	**Mean (SD)**	***P***
**Sex**				0.122		0.577		0.326		0.705
Female	9	1.17	13.10(3.64)		12.82(2.96)		12.44(3.05)		13.88(3.88)	
Male	758	98.83	14.40(2.53)		13.35(2.83)		13.35(2.75)		14.39(2.42)	
**Sex orientation**				0.517		0.871		0.563		0.276
Non-homosexual	55	7.17	14.11(3.25)		13.41(3.22)		13.07(3.62)		13.91(3.37)	
Homosexual	712	92.83	14.40(2.48)		13.33(2.80)		13.36(2.68)		14.42(2.35)	
**Education level completed**				0.117		0.01		0.118		< 0.001
≤High school	175	22.82	14.12(2.57)		12.85(2.94)		13.03(3.11)		13.70(2.62)	
University or above	592	77.18	14.46(2.53)		13.48(2.78)		13.43(2.63)		14.58(2.35)	
**Marital status**				0.549		0.032		0.059		0.035
Unmarried	740	96.48	14.38(2.51)		13.31(2.83)		13.32(2.70)		14.35(2.41)	
Married	27	3.52	14.77(3.36)		14.49(2.86)		14.33(3.56)		15.36(3.01)	
**Body mass index (kg/m** ^**2**^ **)**				0.966		0.239		0.089		0.002
Underweight (< 18.5)	29	3.78	14.29(2.25)		12.51(2.95)		12.240(2.95)		12.874(2.20)^a^	
Normal (18.5–24.9)	508	66.23	14.37(2.61)		13.34(2.85)		13.390(2.74)		14.38(2.49)	
Overweight (≥ 25)	230	29.99	14.41(2.44)		13.45(2.76)		13.370(2.75)		14.57(2.30)	
**Unemployment**				0.038		0.028		0.007		0.081
No	696	90.74	14.45(2.50)		13.42(2.76)		13.45(2.65)		14.44(2.35)	
Yes	71	9.26	13.70(2.89)		12.51(3.33)		12.28(3.45)		13.76(3.17)	
**Income level**,** monthly**				< 0.001		< 0.001		0.002		< 0.001
Low (< NT 40,000)	383	49.93	13.97(2.58)		12.97(2.91)		13.03(2.86)		13.85(2.52)	
High (≥ NT 40,000)	384	50.07	14.79(2.44)		13.71(2.71)		13.65(2.60)		14.91(2.24)	
**Current smoker**				0.115		0.267		0.911		0.272
No	465	60.63	14.50(2.50)		13.43(2.79)		13.35(2.75)		14.46(2.43)	
Yes	298	38.85	14.20(2.61)		13.20(2.90)		13.33(2.77)		14.26(2.47)	
**Alcohol consumption**				0.794		0.913		0.451		0.89
No	429	55.93	14.40(2.54)		13.33(2.89)		13.28(2.87)		14.39(2.53)	
Yes	338	44.07	14.35(2.54)		13.35(2.76)		13.35(2.76)		14.37(2.33)	
**Recreational drugs use history within three months**				0.092		0.253		0.107		0.194
No	609	79.40	14.46(2.50)		13.40(2.77)		13.42(2.68)		14.44(2.40)	
Yes	154	20.08	14.07(2.71)		13.10(3.08)		13.02(2.97)		14.15(2.63)	
**Methamphetamine**				0.142		0.253		0.053		0.112
No	641	83.57	14.44(2.55)		13.39(2.83)		13.43(2.75)		14.44(2.43)	
Yes	125	16.30	14.08(2.51)		13.07(2.83)		12.90(2.75)		14.06(2.47)	
**Sildenafil**				0.024		0.005		0.02		0.015
No	707	92.18	14.44(2.50)		13.42(2.81)		13.41(2.17)		14.44(2.41)	
Yes	59	7.69	13.67(2.90)		12.34(3.01)		12.54(3.08)		13.64(2.71)	
**Having a Lover**				0.243		0.048		< 0.001		0.057
No	447	58.28	14.29(2.56)		13.17(2.89)		13.00(2.83)		14.24(2.49)	
Yes	320	41.72	14.51(2.51)		13.56(2.73)		13.82(2.57)		14.58(2.37)	
**Living with whom**				0.388		0.343		0.108		0.173
Alone	353	46.02	14.25(2.58)		13.19(2.82)		13.12(2.77)		14.20(2.42)	
Family	387	50.46	14.50(2.53)		13.49(2.85)		13.52(2.75)		14.53(2.46)	
Friends	27	3.52	14.39(2.13)		13.16(2.67)		13.74(2.46)		14.58(2.42)	
**Sharing HIV status to**										
**Family members**				0.06		0.928		0.715		0.608
No	422	55.02	14.54(2.43)		13.33(2.73)		13.37(2.75)		14.42(2.35)	
Yes	345	44.98	14.19(2.66)		13.35(2.96)		13.30(2.76)		14.33(2.55)	
**Friends**				0.49		0.984		0.093		0.863
No	715	93.22	14.36(2.56)		13.34(2.85)		13.30(2.77)		14.38(2.46)	
Yes	52	6.78	14.62(2.36)		13.35(2.53)		13.96(2.39)		14.44(2.26)	
**Lovers**				0.462		0.062		< 0.001		0.072
No	270	35.20	14.47(2.48)		13.60(2.70)		13.77(2.61)		14.594(2.38)	
Yes	497	64.80	14.33(2.57)		13.20(2.89)		13.11(2.80)		14.262(2.47)	
**Depressive symptoms (CESD** ≥ **16)**				< 0.001		< 0.001		< 0.001		< 0.001
No	472	61.54	15.52(1.97)		14.72(2.12)		14.48(2.24)		15.31(2.03)	
Yes	295	38.46	12.55(2.27)		11.13(2.39)		11.53(2.51)		12.89(2.31)	
**Hepatitis B virus carrier**				0.252		0.481		0.767		0.864
No	711	92.70	14.35(2.53)		13.32(2.84)		13.33(2.74)		14.38(2.41)	
Yes	56	7.30	14.76(2.68)		13.60(2.75)		13.45(2.92)		14.33(2.81)	
**Hepatitis C virus carrier**				0.108		0.588		0.516		0.158
No	712	92.83	14.42(2.53)		13.35(2.84)		13.36(2.77)		14.41(2.42)	
Yes	55	7.17	13.85(2.65)		13.14(2.72)		13.11(2.56)		13.93(2.63)	
**History of sexual transmitted disease acquired**				0.657		0.16		0.145		0.204
No	273	35.59	14.33(2.69)		13.15(2.95)		13.15(2.86)		14.23(2.55)	
Yes	494	64.41	14.41(2.46)		13.45(2.76)		13.45(2.69)		14.46(2.38)	
**History of syphilis**				0.414		0.052		0.052		0.111
No	338	44.07	14.30(2.62)		13.11(2.94)		13.12(2.83)		14.22(2.51)	
Yes	429	55.93	14.45(2.48)		13.52(2.74)		13.51(2.68)		14.50(2.38)	
**History of gonorrhea**				0.063		0.273		0.273		0.143
No	681	88.79	14.44(2.53)		13.38(2.84)		13.38(2.71)		14.43(2.44)	
Yes	86	11.21	13.90(2.60)		13.02(2.77)		13.03(3.10)		14.02(2.41)	
**History of genital warts**				0.868		0.856		0.402		0.707
No	638	83.18	14.37(2.56)		13.33(2.86)		13.30(2.77)		14.364(2.47)	
Yes	129	16.82	14.41(2.48)		13.38(2.67)		13.53(2.67)		14.451(2.29)	
**CD4 count**,** cells/mm3**				0.618		0.466		0.848		0.974
<200	7	0.91	14.857(1.19)		13.71(1.27)		13.14(3.02)		14.35(1.11)	
≥200	760	99.09	14.376(2.55)		13.34(2.84)		13.34(2.75)		14.38(2.45)	
**Adherence to ART**				0.026		0.008		0.065		0.006
Adherence (MARS ≥ 23)	702	91.53	14.43(2.58)		13.41(2.86)		13.40(2.76)		14.45(2.43)	
Non-adherence (MARS < 23)	65	8.47	13.83(2.00)		12.56(2.36)		12.74(2.62)		13.58(2.43)	
**STR**				0.827		0.859		0.448		0.776
No	142	18.51	14.42(2.32)		13.30(2.98)		13.50(2.74)		14.43(2.37)	
Yes	625	81.49	14.37(2.50)		13.35(2.80)		13.31(2.76)		14.37(2.46)	
**Total participants QoL**	**767**	**100**	**14.38(2.54)**		**13.34(2.83)**		**13.34(2.75)**		**14.38(2.44)**	

ART, Antiretroviral therapy; CES-D, Center for Epidemiologic Studies Depression Scale; MARS, Medication Adherence Report Scale; PWH, people with HIV; SD, standard deviation; STR, single table regimen

^a^ Mean difference between groups by post hoc analysis

The CES-D scale, a 20-item validated self-report questionnaire, measures depressive symptoms, including mood disturbances, appetite changes, sleep quality, and concentration difficulties [13]. A score ≥ 16 suggests significant depressive symptoms requiring further clinical evaluation. The CES-D demonstrated good internal consistency in this study (Cronbach’s α = **0.842**).

The MARS-5 scale, widely used for medication adherence assessment, includes five Likert-scale questions (scores range from 5 to 25) and is utilized in previous PWH’s study in Taiwan [14]. A score < 23 indicates non-adherence to ART. The MARS-5 showed acceptable internal consistency in this sample (Cronbach’s α = **0.769**).

### Outcomes

The primary aim of this research was to evaluate the participants’ quality of life and identify the factors that impact it.

### Statistical analysis

The results for categorical variables were presented as frequencies and percentages (n, %), while those for continuous variables were summarized as means and standard deviations (SD). Differences across quality-of-life domains based on various socio-demographic variables were evaluated using T-tests for comparisons between two groups or ANOVA for comparisons among more than two groups, as appropriate.

Linear regression analysis was performed to examine the association between socio-demographic variables and quality of life. Dummy variables were created for categorical variables with more than two categories. Variables with a p-value < 0.1 in univariate analyses were included in the multivariate linear regression model to adjust for confounding factors. The model applied a stepwise elimination process to refine variable selection. A stepwise variable selection approach was applied due to the exploratory nature of the study and the large number of potential sociodemographic and clinical predictors. Multicollinearity was assessed using variance inflation factors (VIF), with all VIF values < 3, indicating a low risk of multicollinearity. Statistical significance was determined using a two-tailed p-value < 0.05. All statistical analyses were conducted using SPSS software, version 22.0 (SPSS Inc., Chicago, IL, USA).

## Results

### Characteristics of virally suppressed PWH and their quality of life

Between December 2018 and June 2021, a total of 767 individuals living with HIV who had undetectable viral loads were recruited for analysis. The mean age of participants was 37.9 years (standard deviation [SD] = 8.89), with an average duration of HIV diagnosis of 9.25 years (SD = 5.50). The mean ART treatment duration was 7.97 years (SD = 5.54). Regarding sex and sexual orientation, 758 individuals (98.83%) identified as male, while 9 (1.17%) identified as female. Most participants identified as homosexual (*n* = 712, 92.83%).

QoL scores varied across domains, with the highest scores observed in the environmental (14.38, SD = 2.44) and physical (14.38, SD = 2.54) dimensions, while the psychological (13.34, SD = 2.83) and social (13.34, SD = 2.75) domains scored slightly lower. Across various QoL domains, higher education, marital status, income levels, having a romantic partner, and greater ART adherence were positively associated with higher QoL scores. Conversely, depressive symptoms, unemployment, underweight status, and history of sildenafil use were linked to lower QoL outcomes (Table 1).

**Table 2:** Self-perceived quality of life and health conditions

		Self-perceived health condition (Q2), score				
		1.00	2.00	3.00	4.00	5.00
**Self-perceived QoL (Q1)**,** score**	**1.00**	5	3	1	1	**0**
	**2.00**	4	38	14	**4**	**0**
	**3.00**	3	94	**194**	**58**	**2**
	**4.00**	2	**15**	**106**	**163**	**8**
	**5.00**	**0**	**1**	**9**	**24**	**18**

Bold font represents Q1 + Q2 scores ≥ 6, indicating good QoL. A total of 602 participants (78.5%) reported good QoL

Note: Domain scores range from 1 to 10, with higher scores indicating better QoL. In line with Silva et al. (2014) [15], a cut-off of ≥ 6 points was adopted to represent “good” QoL. This threshold was originally validated in older adults, not in people living with HIV (PWH), but was applied here due to the absence of HIV-specific cut-offs

### Self-perceived quality of life and health conditions

Participants’ self-perceived QoL and health satisfaction were assessed by the first two questions in the WHOQOL-BREF questions. Applying a cutoff score of ≥ 60, based on previous study (Q1 + Q2 scores ≥ 6) [15], 602 participants (78.5%) reported good QoL, while 21.5% fell below this threshold, indicating significant challenges in overall well-being (Table 2).

### Factors associated with participant characteristics and QoL

Univariate linear analysis was used to analyze the participant characteristics associated with each QoL domain. Physical, psychological, social, and environmental QoL were negatively associated with depressive symptoms, unemployment, and a history of sildenafil use, but were positively associated with higher income levels. A university education or above and being married were linked to better psychological and environmental QoL, while non-adherence to ART was associated with poorer QoL in these domains. Regarding body weight, participants with normal or overweight statuses tended to have better social and environmental QoL. Having a romantic partner was linked to better QoL in the psychological and social domains, whereas disclosing HIV status to one’s partner was associated with poorer QoL in the social domain. Additionally, living with family members was associated with better social QoL (Table 3).

**Table 3:** Univariate linear analyses of relationship between PWH characteristics and QoL

Socio-demographics	Physical		Psychological		Social		Environmental	
	Regression coefficient (standard errors)	*P*	Regression coefficient (standard errors)	*P*	Regression coefficient (standard errors)	*P*	Regression coefficient (standard errors)	*P*
**Age**,** yr**	0.005(0.010)	0.632	0.010(0.012)	0.393	-0.012(0.011)	0.289	0.009(0.010)	0.378
**Diagnosed**,** yr**	-0.018(0.017)	0.279	-0.009(0.019)	0.625	-0.012(0.018)	0.497	-0.001(0.016)	0.962
**Treatment duration**,** yr**	-0.005(0.017)	0.747	0.007(0.018)	0.713	-0.004(0.018)	0.831	0.006(0.016)	0.724
**Sex**								
Female	1		1		1		1	
Male	1.316(0.851)	0.122	0.530(0.950)	0.577	0.908(0.923)	0.326	0.509(0.819)	0.535
**Sex orientation**								
Non-homosexual	1		1		1		1	
Homosexual	0.292(0.356)	0.412	-0.073(0.396)	0.854	0.290(0.385)	0.452	0.509(0.341)	0.137
**Education level completed**								
≤High school	1		1		1		1	
University or above	0.343(0.218)	0.117	0.629(0.243)	0.01	0.406(0.237)	0.087	0.881(0.208)	< 0.001
**Marital status**								
Unmarried	1		1		1		1	
Married	0.406(0.498)	0.415	1.197(0.553)	0.031	1.028(0.538)	0.057	1.014(0.477)	0.034
**Body mass index (kg/m** ^**2**^ **)**								
Underweight (< 18.5)	1							
Normal (18.5–24.9)	0.088(0.486)	0.857	0.830(0.540)	0.125	1.152(0.524)	0.028	1.504(0.463)	0.001
Overweight (≥ 25)	0.122(0.501)	0.808	0.944(0.558)	0.091	1.124(0.541)	0.038	1.698(0.478)	< 0.001
**Unemployment**								
No	1		1		1		1	
Yes	-0.752(0.316)	0.017	-0.916(0.351)	0.009	-1.168(0.341)	0.001	-0.684(0.303)	0.025
**Income level**,** monthly**								
Low (< NT 40,000)	1		1		1		1	
High (≥ NT 40,000)	0.812(0.181)	< 0.001	1.056(0.324)	0.001	1.271(0.313)	< 0.001	1.206(0.278)	< 0.001
**Current smoker**								
No	1		1		1		1	
Yes	-0.300(0.183)	0.102	-0.227(0.204)	0.267	-0.008(0.199)	0.968	-0.202(0.176)	0.252
**Alcohol consumption**								
No	1		1		1		1	
Yes	-0.048(0.185)	0.794	0.022(0.206)	0.913	0.151(0.200)	0.451	-0.025(0.178)	0.89
**Recreational drugs use history within three months**								
No	1		1		1		1	
Yes	-0.235(0.262)	0.37	-0.183(0.292)	0.531	-0.240(0.284)	0.399	-0.202(0.252)	0.422
**Methamphetamine**								
No	1		1		1		1	
Yes	-0.365(0.244)	0.135	-0.284(0.272)	0.298	-0.494(0.264)	0.062	-0.378(0.235)	0.107
**Sildenafil**								
No	1		1		1		1	
Yes	-0.748(0.332)	0.025	-0.969(0.370)	0.009	-0.788(0.360)	0.029	-0.776(0.319)	0.015
**Having a Lover**								
No	1		1		1		1	
Yes	0.218(0.186)	0.243	0.409(0.207)	0.048	0.819(0.199)	< 0.001	0.341(0.178)	0.057
**Living with whom**								
Alone	1		1		1		1	
Family	0.258(0.187)	0.169	0.297(0.208)	0.155	0.398(0.202)	0.0495	0.327(0.179)	0.069
Friends	0.146(0.507)	0.773	-0.029(0.565)	0.959	0.622(0.549)	0.257	0.384(0.487)	0.431
**Sharing HIV status to**								
**Family members**								
No	1		1		1		1	
Yes	-0.350(0.184)	0.058	0.019(0.206)	0.928	-0.073(0.200)	0.715	-0.091(0.177)	0.608
**Friends**								
No	1		1		1		1	
Yes	0.252(0.365)	0.49	0.008(0.407)	0.984	0.665(0.395)	0.093	0.061(0.351)	0.863
**Lovers**								
No	1		1		1		1	
Yes	-0.141(0.192)	0.462	-0.400(0.214)	0.062	-0.667(0.207)	0.001	-0.332(0.184)	0.072
**Depression symptoms (CES-D** ≥ **16)**								
No	1		1		1		1	
Yes	-2.971(0.155)	< 0.001	-3.593(0.165)	< 0.001	-2.951(0.174)	< 0.001	-2.415(0.159)	< 0.001
**Hepatitis B virus carrier**								
No	1		1		1		1	
Yes	0.404(0.353)	0.252	0.277(0.393)	0.481	0.113(0.382)	0.767	-0.058(0.339)	0.864
**Hepatitis C virus carrier**								
No	1		1		1		1	
Yes	-0.572(0.355)	0.108	-0.215(0.396)	0.588	-0.250(0.385)	0.516	-0.483(0.341)	0.158
**History of sexual transmitted disease acquired**								
No	1		1		1		1	
Yes	0.085(0.192)	0.657	0.300(0.213)	0.16	0.303(0.207)	0.145	0.234(0.184)	0.204
**History of syphilis**								
No	1		1		1		1	
Yes	0.151(0.185)	0.414	0.401(0.206)	0.052	0.389(0.200)	0.052	0.283(0.177)	0.111
**History of gonorrhea**								
No	1		1		1		1	
Yes	-0.541(0.290)	0.063	-0.355(0.324)	0.273	-0.345(0.315)	0.273	-0.410(0.279)	0.143
**History of genital warts**								
No	1		1		1		1	
Yes	0.041(0.245)	0.868	0.050(0.273)	0.856	0.223(0.266)	0.402	0.089(0.236)	0.707
**CD4 count**,** cells/mm3**								
<200	1		1		1		1	
≥200	-0.418(0.965)	0.618	-0.379(1.076)	0.725	0.201(1.046)	0.848	0.030(0.927)	0.974
**Adherence to ART**								
Adherence (MARS ≥ 23)	1		1		1		1	
Non-adherence (MARS < 23)	-0.603(0.329)	0.067	-0.846(0.366)	0.021	-0.659(0.356)	0.065	-0.874(0.315)	0.006
**STR**								
No	1		1		1		1	
Yes	-0.052(0.236)	0.827	0.047(0.263)	0.859	-0.194(0.256)	0.448	-0.065(0.227)	0.776

ART, Antiretroviral therapy; CES-D, Center for Epidemiologic Studies Depression Scale; MARS, Medication Adherence Report Scale; PWH, people with HIV; QoL, quality of life; STR, single table regimen

Multiple linear regression analyses were conducted to determine the key factors influencing the QoL of virally suppressed PWH. After adjusting for demographic characteristics and other covariates, depressive symptoms remained the strongest negative predictor of QoL across all domains (Model 1). On average, participants with significant depressive symptoms scored approximately 2–3 points lower in each WHOQOL-BREF domain compared to those without symptoms. Given that mean domain scores in this population ranged between 13 and 14 (Table 1), this reduction represents a 20–25% decline in perceived quality of life, indicating a clinically meaningful impairment in daily functioning and well-being. Model 2 examined the effects of individual domains in detail. Beyond depressive symptoms, higher income was associated with increases of 0.38–0.59 points in physical and environmental QoL, reflecting the role of economic stability in healthcare access and living conditions. Marital status was associated with 1.09–1.25 point higher psychological and environmental QoL, suggesting that spousal support provides emotional stability and reduces isolation. Higher educational attainment was associated with 0.45–0.62 point higher psychological and environmental QoL, likely through improved health literacy and coping capacity. Having a romantic partner was related to a 0.54 point increase in social QoL, underscoring the protective role of intimate relationships in mitigating stigma and fostering social support. Although these effect sizes were smaller than those observed for depressive symptoms, they remain clinically meaningful, highlighting the tangible contributions of socioeconomic and relational factors to QoL among virally suppressed PWH (Table 4).

**Table 4:** Multiple linear regression analysis of relationship between participants characteristics and quality of life

	Physical		Psychological		Social		Environmental	
	Regression coefficient (standard errors)	*P*	Regression coefficient (standard errors)	*P*	Regression coefficient (standard errors)	*P*	Regression coefficient (standard errors)	*P*
**Model 1**								
Depression symptoms (ref: no)	-2.971(0.155)	< 0.001	-3.593(0.165)	< 0.001	-2.951(0.174)	< 0.001	-2.302(0.159)	< 0.001
adjusted R^2^	0.323		0.381		0.272		0.231	
**Model 2**								
Depression symptoms (ref: no)	-2.911(0.157)	< 0.001	-3.356(0.164)	< 0.001	-2.898(0.174)	< 0.001	-2.290(0.157)	< 0.001
Income (ref: low)	0.376(0.152)	0.014					0.588(0.157)	< 0.001
Marital status (ref: unmarried)			1.246(0.436)	0.04			1.087(0.413)	0.009
Education level completed (ref: ≤High school)			0.453(0.192)	0.018			0.622(0.186)	0.001
Having a Lover (ref: no)					0.538(0.172)	0.002		
adjusted R^2^	0.328		0.389		0.28		0.265	

## Discussion

This study examines the quality of life among virally suppressed PWH in northern Taiwan, identifying key determinants that influence their well-being across physical, psychological, social, and environmental domains. These determinants include depressive symptoms, socioeconomic factors such as income and education, marital status, and being in a relationship. Compared with community-dwelling elderly [16], healthy workers [17], and sexual and gender minorities in Taiwan, PWH reported significantly lower QoL scores in all domains. As Taiwan progresses toward achieving the 95-95-95 UNAIDS targets and embraces the Fourth 90, our findings underscore the need to shift from survival to holistic health, ensuring that PWH not only live longer but also experience a fulfilling quality of life.

Depressive symptoms emerge as the strongest negative predictor of poorer QoL across physical health, psychological well-being, social relationships, and environmental conditions, as previously observed in studies conducted in Taiwan [18, 19]. In our sample, depressive symptoms have lower QoL scores by ~ 2–3 points across domains, representing a 20–25% decline relative to mean scores. This magnitude is clinically meaningful, reflecting substantial impairment in daily functioning and well-being. The prevalence of depressive symptoms among PWH is approximately 23.7% in Taiwan [20] and 50.8% in China [21], which significantly higher than those observed in the general population [22, 23]. Hou et al. identified depressive symptoms as the most significant negative predictor of QoL, while social support and early initiation of ART were positively correlated with QoL. PWH often faces stigma, discrimination, and social isolation, all of which are well-documented contributors to mental health challenges [24]. Additionally, HIV-associated neuroinflammation may exacerbate depressive symptoms, particularly among long-term ART users [25]. Both internal and external factors contribute to the heightened prevalence of depressive symptoms among PWH, making mental health a significant barrier to HIV care and treatment adherence [26, 27]. These findings highlight the importance of integrating mental health support into HIV management programs to ensure both psychological well-being and sustained treatment success. Interventions such as cognitive-behavioral therapy (CBT), peer support groups, and collaborative care models involving psychologists and HIV specialists could provide valuable support [28], helping to break the cycle of poor mental health and inadequate treatment adherence.

Economic stability is crucial in shaping QoL outcomes for PWH, as noted in this study. Higher income is significantly associated with better physical and environmental QoL, mirroring findings from studies in other regions [8, 29]. Economic security enhances access to better nutrition, healthcare services, and stable living conditions, which contribute to overall well-being. Additionally, our findings align with previous research showing that PWH with higher educational attainment report better psychological and environmental QoL [30]. Education is often linked to greater health literacy, enhanced coping mechanisms, and stronger social networks, all of which foster resilience among PWH. Moreover, a higher level of education enables access to better job opportunities and career choices, leading to improved living conditions. These factors logically correspond to higher scores in environmental QoL, as observed in a previous study conducted in Taiwan [31].

Marriage also emerges as a positive predictor of psychological and environmental QoL for PWH, primarily due to the emotional support provided by spouses, financial stability, and its protective effects against loneliness and social isolation [32–34]. Moreover, PWH in romantic relationships tend to achieve higher social QoL scores, likely because their partners provide crucial social support [35]. This support helps mitigate the negative effects of HIV-related stressors and stigma, fostering stronger social connections and enhancing overall social well-being. However, stigma remains a significant challenge for many PWH seeking intimate relationships, often resulting in social exclusion and mental distress [36]. In Taiwan, where PWH are predominantly members of the LGBTQ + community, the legalization of same-sex marriage in 2019 marked a significant step toward equality [37]. Yet our findings suggest that societal stigma continues to undermine these potential QoL gains. Despite this legislative progress, societal discrimination persists, hindering the ability of PWH to form stable relationships and fully benefit from marriage’s protective effects. Research indicates that while legal recognition has improved rights for same-sex couples, stigma surrounding HIV and LGBTQ + identities continues to restrict social acceptance and access to family support [38]. For example, some PWH report being denied equal treatment in medical settings, facing workplace exclusion after disclosing HIV status, or experiencing rejection from family members despite legal recognition of their partnerships. While marriage can enhance psychological and environmental QoL for PWH, overcoming persistent stigma in Taiwan requires more than just legislative change. Legal advancements must be accompanied by broader cultural shifts that foster genuine acceptance and inclusive family and community support. Addressing these barriers through education, policy initiatives, and anti-discrimination measures is essential for creating a more equitable and supportive society for PWH.

Although sildenafil use showed a negative association with QoL in the univariate analysis, this association did not remain significant in the multivariable model. This pattern suggests that sildenafil use may reflect underlying factors—such as sexual dysfunction, psychological distress, or relationship-related stressors—rather than exerting an independent effect on QoL. Prior studies among men living with HIV have reported that sexual difficulties are linked to poorer mental health and lower health-related QoL [39], indicating that sildenafil use may act as a proxy for these broader psychosocial challenges rather than a causal determinant. Reviews of sexual dysfunction in men with HIV further highlight that erectile difficulties arise from multifactorial contributors, including HIV-related inflammation, antiretroviral therapy effects, comorbidities, body image concerns, and internalized stigma [40]. Because the study did not collect detailed information on the indications for sildenafil use, residual confounding or reverse causation cannot be ruled out. Future studies incorporating validated measures of sexual function may help clarify these pathways.

This study found that Taiwan’s Fourth 90 target for HIV care to be around 78.5%, indicating significant unmet needs. To achieve QoL parity with viral suppression goals, several critical implications for HIV care providers and policymakers should be considered. Firstly, integration of mental health into HIV treatment is essential. Routine screenings for depressive symptoms using validated tools such as CES-D should be incorporated into HIV care settings to identify and manage psychological distress early. Further interventions, including cognitive-behavioral therapy, peer support groups, and psychiatric care, could improve the QoL of PWH facing mental health challenges. Secondly, social and economic support systems must be strengthened. Since income and education significantly impact QoL, policymakers should expand employment training, financial assistance programs, and educational opportunities tailored for PWH. Moreover, relationship and marital support should be prioritized, as having a committed partner or spouse enhances QoL by providing emotional stability and social support. Policymakers should cultivate an inclusive environment that reduces stigma, ensuring PWH have equal opportunities in relationships and marriage. By integrating mental health care, strengthening social and economic support, and fostering relationship-friendly policies, Taiwan can move closer to achieving the Fourth 90 goal in QoL for PWH.

Despite its valuable contributions, this study has several limitations. First, participants were recruited from a single hospital-based HIV clinic in Taipei, which may limit the generalizability of our findings to other regions or healthcare settings in Taiwan. Second, the sample was overwhelmingly male (98.8%) and predominantly MSM (92.8%), reflecting the epidemiology of HIV in Taiwan but limiting generalizability to women, heterosexual PWH, and individuals receiving care in non-urban or community-based settings. Future studies should include more diverse populations to enhance representativeness. Third, the cross-sectional design precludes causal inference regarding the relationships between potential determinants and QoL; longitudinal studies are warranted to clarify temporal dynamics and causal pathways. Fourth, the reliance on self-reported measures may have introduced information bias. Depressive symptoms and medication adherence were assessed through self-report, which may be subject to recall bias, social desirability bias, or underreporting due to HIV-related stigma. Fifth, while the WHOQOL-BREF is a validated instrument for assessing QoL among PWH, it does not explicitly capture stigma-related experiences such as discrimination or internalized shame, which are highly relevant to this population. Finally, the cut-off value of ≥ 6 points used to classify “good” self-perceived QoL was derived from a study of older adults rather than from HIV-specific populations. Although this threshold served as a pragmatic reference for descriptive interpretation, caution is necessary when extrapolating this cut-off to people with HIV. Future studies should aim to establish HIV-specific and culturally appropriate QoL cut-off values to enhance clinical interpretability and contextual relevance in Taiwan and other settings.

## Conclusions

This study provides comprehensive insights into the QoL determinants among virally suppressed PWH in Taiwan, reinforcing the need for holistic, patient-centered HIV care approaches. While Taiwan has made significant progress in achieving Triple 95 targets, future efforts must integrate mental health care, socioeconomic support, stigma reduction initiatives, and relationship support into HIV policy frameworks. By addressing psychological, social, and environmental factors alongside medical treatment, healthcare providers and policymakers can ensure that PWH not only survive but thrive—living with dignity, stability, and optimal well-being.

## Data Availability

The datasets used and/or analyzed during the current study are available from the corresponding author on reasonable request due to privacy and ethical considerations.

## Abbreviations

AIDS: Acquired immunodeficiency syndrome
ART: Antiretroviral therapy
CBOs: Community-based organizations
CBT: Cognitive-behavioral therapy
CDC: Centers for disease control
CES-D: Center for epidemiologic studies depression scale
GHB: Gamma-hydroxybutyrate
MARS-5: Medication adherence report scale
PrEP: Pre-exposure prophylaxis
PWH: People with HIV
QoL: Quality of life
SD: Standard deviations
STIs: Sexually transmitted infections
UNAIDS: The Joint United Nations Programme on HIV/AIDS
U = U: Undetectable equals untransmittable
VIF: Variance inflation factors
WHOQOL-BREF: World health organization quality of life scale - brief version
